# Chromosomal instability in the lymphocytes of breast cancer patients

**DOI:** 10.4103/0971-6866.50864

**Published:** 2009

**Authors:** Kaur Harsimran, Monga Gaganpreet Kaur, Setia Nitika, Sudan Meena, Uppal M. S., Yamini, Batra A. P. S., Sambyal Vasudha

**Affiliations:** 1 Department of Human Genetics, Guru Nanak Dev University, Amritsar, Punjab-143 005, India; 2 Department of Surgery, Sri Guru Ram Das Institute of Medical Sciences and Research (SGRDIMSR), Amritsar, Punjab-143 005, India; 3 Department of Anatomy, Sri Guru Ram Das Institute of Medical Sciences and Research (SGRDIMSR) Amritsar, Punjab-143 005, India

**Keywords:** Breast cancer, chromosomal aberrations, genomic instability, lymphocytes

## Abstract

Genomic instability in the tumor tissue has been correlated with tumor progression. In the present study, chromosomal aberrations (CAs) in peripheral blood lymphocytes (PBLs) of breast tumor patients were studied to assess whether chromosomal instability (CIN) in PBLs correlates with aggressiveness of breast tumor (i.e., disease stage) and has any prognostic utility. Cultured blood lymphocyte metaphases were scored for aberrations in 31 breast cancer patients and 20 healthy age and sex-matched controls. A variety of CAs, including aneuploidy, polyploidy, terminal deletions, acentric fragments, double minutes, chromatid separations, ring chromosome, marker chromosome, chromatid gaps, and breaks were seen in PBLs of the patients. The CAs in patients were higher than in controls. A comparison of the frequency of metaphases with aberrations by grouping the patients according to the stage of advancement of disease did not reveal any consistent pattern of variation in lymphocytic CIN. Neither was any specific chromosomal abnormality found to be associated with the stage of cancer. This might be indicative of the fact that cancer patients have constitutional CIN, which predisposes them to the disease, and this inherent difference in the level of genomic instability might play a role in disease progression and response to treatment.

## Introduction

Cancer is a complex disease in which cells with altered gene expression grow abnormally, invade other tissues, and disrupt their normal function. A crucial early event in carcinogenesis is the induction of the genomic instability phenotype, which enables an initiated cell to evolve into a cancer cell by achieving greater proliferative capacity and genetic plasticity to overcome host immunological resistance, localized toxic environment, and suboptimal micronutrient supply.

Genomic instability in cancer can be of two types: microsatellite instability (MIN) and chromosomal instability (CIN). MIN tumors exhibit an apparently normal karyotype and have mutations in DNA mismatch repair genes. But, a majority of the tumors exhibit abnormal karyotypes involving either chromosomal rearrangement and/or aneuploidy and are classified as CIN tumors.[1–3]

Various reports indicate a significant increase in the chromosomal aberrations (CAs) in peripheral blood lymphocytes (PBLs) of cancer patients with solid tumors.[4–7] PBLs of patients with breast cancer and other solid tumors show simple chromosomal lesions that may be stable markers in cancer cells.[8] Hence, it is proposed that lymphocytes may be used as a surrogate tissue model for studying genomic instability in case of solid tumors and the frequency of CAs in PBLs can be used as a predictor of cancer risk.[9–12]

Breast cancer is a major global health problem and the incidence of the disease continues to increase steadily. The frequency of sporadic breast cancer is higher in areas adjoining Amritsar city of Punjab, India (Unpublished data, Rotary Cancer Hospital, Amritsar; personal comunication). In the present study, CAs in PBLs of sporadic breast tumor patients were studied to assess whether CIN in PBLs correlates with aggressiveness of breast tumor, i.e. disease stage, and has any prognostic utility.

## Materials and Methods

Five milliliters of blood sample from 31 cancer patients, 28 with sporadic malignant breast cancer and three with benign breast disease, were collected before surgery from the surgical wards of Sri Guru Ram Das Institute of Medical Sciences and Research, Amritsar (Punjab), after informed consent was obtained. Institutional ethical committee approval was obtained for the study. Relevant information regarding age, symptoms, duration and stage of the disease (TNM classification), habits, habitat, menstrual and reproductive history, occupation, and exposure of the patients to mutagens was recorded on a predesigned questionnaire. Age and sex-matched controls were randomly selected from the general population of Amritsar. Blood samples of 31 breast cancer patients and 20 healthy age and sex-matched controls were cultured in RPMI 1640 medium according to the standard culturing technique,[13] with some modifications. Slides were GTG banded according to the Benn and Perle[14] method. Banded slides were scanned for numerical and structural aberrations. For each subject, 100 clear metaphases were assessed for CAs. Of these, 10 metaphases were karyotyped as per ISCN 2005. The *t*-test was used to compare the frequency of aberrant metaphases among patients and controls.

## Results

Among the patients, one patient had stage IV disease, 13 had stage III disease, eight were diagnosed at stage II, six had stage I, and three patients had benign disease. The cancer patients were in the age group of 28–65 years. None of the patients had history of early menarche (before the age of 12 years) or late menopause (after 55 years) in postmenopausal patients. 77.4% of the patients (*n* = 24) consumed vegetarian diet and only 22.5% (*n* = 7) consumed nonvegetarian food occasionally. All of them had first full-term pregnancy before the age of 30 years. 87.1% (*n* = 27) of the patients were housewives. Twelve patients (38.7%) belonged to urban area, six (19.3%) of them had suburban habitat, and 13 (42%) belonged to rural areas surrounding Amritsar [Table 1]. Control subjects were in the age group of 27–66 years. Fifty-five percent (*n* = 11) of the controls belonged to urban areas, 20% (*n* = 4) of them were from suburban areas, and 25% (*n* = 5) belonged to rural area. Most (90%) (*n* = 18) of them consumed vegetarian diet [Table 1].

**Table 1:** Habits and habitat of the patients and control subjects

Stage	Code	Age	Age at (years)		Habitat	Diet	Occupation
						
			Menarche	Menopause			
Benign	P10	65	15	53	Rural	Veg*	Housewife
	P11	45	16	-	Rural	Veg	Housewife
	P14	60	14	50	Suburban	Veg	Housewife
Stage I	P1	65	15	53	Urban	Veg	Housewife
	P4	45	14	50	Sub urban	Veg	Housemaid
	P6	50	14	45	Urban	Veg	Housewife
	P8	50	15	46	Urban	Veg	Housewife
	P15	35	13	-	Suburban	Veg	Housewife
	P21	50	12	49	Rural	Veg	Housewife
Stage II	P2	60	14	49	Urban	Nonveg†	Housewife
	P3	60	13	45	Urban	Veg	Teacher
	P7	60	15	45	Urban	Veg	Housewife
	P9	65	12	50	Urban	Nonveg	Housewife
	P16	52	14	-	Urban	Nonveg	Teacher
	P18	57	13	50	Suburban	Veg	Housewife
	P22	64	13	55	Rural	Veg	Housewife
	P23	65	13	54	Rural	Veg	Housewife
Stage III	P5	35	13	-	Urban	Veg	Housewife
	P13	42	15	-	Urban	Veg	Housewife
	P17	55	14	50	Suburban	Veg	Housewife
	P19	45	-	45	Rural	Nonveg	Housewife
	P20	48	13	43	Urban	Veg	Housewife
	P24	35	14	-	Rural	Nonveg	Housewife
	P25	28	14	-	Urban	Veg	Housewife
	P26	50	13	-	Rural	Veg	Housewife
	P27	42	14	-	Rural	Veg	Housewife
	P28	65	14	53	Rural	Veg	Housewife
	P29	55	12	53	Suburban	Veg	Housewife
	P30	50	15	-	Rural	Nonveg	Housewife
	P31	47	16	-	Rural	Veg	Housewife
Stage IV	P12	33	14	-	Rural	Nonveg	Laborer
Controls	C1	30	12	-	Urban	Veg	Housewife
	C2	29	14	-	Urban	Nonveg	Teacher
	C3	50	13	45	Urban	Veg	Housewife
	C4	45	12	44	Suburban	Veg	Housewife
	C5	50	13	48	Urban	Veg	Housewife
	C6	28	14	47	Suburban	Veg	Housewife
	C7	60	15	48	Rural	Veg	Housewife
	C8	65	14	46	Rural	Veg	Housewife
	C9	32	12	-	Urban	Veg	Housewife
	C10	42	13	-	Urban	Veg	Teacher
	C11	45	12	44	Urban	Veg	Office job
	C12	53	13	46	Urban	Veg	Office job
	C13	40	12	-	Urban	Veg	Office job
	C14	55	12	47	Urban	Veg	Hostel attendant
	C15	50	13	45	Rural	Veg	Sweeper
	C16	63	14	46	Rural	Veg	Housewife
	C17	53	12	47	Suburban	Veg	Housewife
	C18	66	13	46	Rural	Veg	Farmer
	C19	60	11	47	Suburban	Veg	Housewife
	C20	27	13	-	Urban	Nonveg	Student

* Vegeterian

† Nonvegeterian

The frequency of aberrant metaphases varied from 3.3 to 60.1% in cultured lymphocytes of patients and from 1.5 to 5.7% in controls [Table 2]. Stage I patients had aberrant metaphases ranging from 21.4 to 60%. The frequency of aberrant metaphases ranged from 20 to 50% in stage II patients and 3.3 to 60% in stage III and IV patients. Enormous variation was also seen for numerical and structural aberrations among patients with various stages of advancement of disease. A variety of CAs, including aneuploidy, polyploidy, terminal deletions, acentric fragments, double minutes, chromatid separations, ring chromosome and marker chromosome, chromatid gaps, and breaks were seen in PBLs of the patients. Specific CA correlated with stage of cancer was not observed, neither was any particular chromosome found to be involved in aberrations in all the patients. A high frequency of acrocentric associations (D-D, G-G, D-G) was seen in all the patients as compared to controls. The mean value of percent total aberrations in patients was 32.3% and in control subjects was 1.9%. A statistically significant difference in the percentage of aberrant metaphases was seen among patients and controls (*t*-value = 10.1, *P* < 0.001).

**Table 2:** Comparison of frequency (%age) of aberrant metaphases in peripheral blood lymphocytes of breast cancer patients (grouped according to disease stage) and controls

Category	Mean age (years)	Mean age (years) at		Mps‡ with total aberrations (%)		Mps with numerical aberration (%)		Mps with structural aberration (%)		Mps with acrocentric association (%)	
						
		Menarche	Menopause	Mean ± SD	Range	Mean± SD	Range	Mean ± SD	Range	Mean ± SD	Range
Benign (*n* = 3)	56.7	15	51.5	20.2	15.7-23.3	16.7	7.9-23.3	4.4	5.2-8.11	28.6	27.02-28.9
Stage I (*n* = 6)	49.2	13.8	48.6	40.4	21.4-60	29.9	12.3-50	14.9	3.6-26	13.0	2.5-31.2
Stage II (*n* = 8)	60.4	13.4	49.7	33.7	20-50	25.9	16.6-24	14.4	0-27.8	9.4	2.5-25.2
Stage III (*n* = 13)	45.9	9.4	48.8	30.8	3.3-60.1	22.8	3.3-68	15.4	0-30.2	12.9	3.3-25.7
Stage IV (*n* = 1)	33	14	-	27.27		12.1	-	15.15	-	18.18	-
Statistical comparison of chromosomal aberrations using *t*-test											
Patients (*n* = 31)				32.3±13.0	3.3-60.1	24.1±13.2	3.3-68.0	15.5±8.2	0-30.2	13.8±10.3	2.5-35.7
Controls (*n* = 20)				1.9±2.1	0-5.7	2.6±1.3	0-5.0	3.9±0.9	0-5.1	4.3±2.6	0-11.1
Statistical significance				Significant difference (*P* < 0.0001)		Significant difference (*P* < 0.0001)		Significant difference (*P* < 0.005)		Significant difference (*P* < 0.005)	

‡ Metaphases

## Discussion

Genetic instability is a defining feature of human cancer. In the present study, breast cancer patients had a significantly higher percentage of aberrant metaphases as compared with controls. There was a high frequency of numerical as well as structural abnormalities in the cultured lymphocytes of patients, but enormous variation was seen in the level of lymphocytic CIN among the breast cancer patients. The mean of percentage of metaphases with aberrations was 20.2% in patients with benign disease, 40.4% in stage I patients, 33.9% in stage II patients, 30.8% in stage III patients, and 27.3% in stage IV patients. However, the percentage of aberrant metaphases ranged from 15.7 to 23.3% in patients with benign disease, 21.4 to 60% in stage I patients, 20.1 to 50.2% in stage II patients, and 3.3 to 60.1% in stage III patients, suggestive of variability in the underlying genomic composition of these patients. Grouping of patients according to the stage of advancement of disease did not reveal any consistent pattern of variation in lymphocytic CIN [Table 2], in contrast to tumor tissue where genomic instability has been correlated with tumor progression. Genomic instability has been found to be low in benign and hyperplastic tissues, but dramatically increased in ductal carcinoma and invasive cancer.[15] Frequency of allelic imbalance (or MIN) in tumor tissue has been shown to be significantly correlated with tumor progression in colorectal cancer.[16] In a fluorescent *in situ* hybridization study of numerical alterations of chromosomes 7, 8, 16, and 17 in 28 ductal carcinoma in situ (DCIS), it was shown that the patterns of aneuploidy in breast tumor tissue may differ according to the tumor grade.[17] This indicated that lymphocytic CIN was an index of inherent instability in the patient′s genome and was not influenced by the disease status, whereas genomic instability of the tumor could be influenced by the patient′s disease status or aggressiveness of tumor.

High frequency of aberrations in PBLs of breast cancer patients similar to that seen in tumor tissue has already been reported in several studies.[12,18–20] Also, greater than expected infrared-induced genomic instability has been seen in lymphocytes of patients with breast cancer and other solid tumors.[21–23] Thus, cancer patients probably have constitutional CIN, which participates in cancer predisposition.

Aberrations involving specific chromosomes (2, 7, 11, 12, 15, 19, 22, and X) in the lymphocytes of breast cancer patients have been reported in a previous study.[20] Various genes involved in genomic stability and breast tumorigenesis [EP300 (22q13.2), LKB1 (19p13.3), FGFR1 (8p11.2), CHEK2 (22q) and K-ras (12p12)] are located in these regions and might be involved in the variable CIN phenotype. The variable CIN phenotype is due to alterations at different CIN loci. CIN genes are involved in a variety of pathways, including chromosome condensation, sister chromatid cohesion, kinetochore structure and function, microtubule formation, and cell-cycle control.[24]

Another interesting observation from the analysis of epidemiological data of the patients was that many of the well-established epidemiological risk factors reported in previous studies on western data (i.e., late age of menopause, early age of menarche, nulliparity, older age at first birth, alcohol consumption, high meat intake, and high socioeconomic status[25,26]) did not account for the etiology of the disease in patients in the present study. Early age at menarche (less than 12 years of age) has been associated with a 10-20% increase in breast cancer risk and delayed menopause (after 54 years of age) maximizes the number of ovulatory cycles, leading to increased breast cancer risk.[27–29] Nulliparity and late age at first birth also contribute toward an increased risk of developing breast cancer.[25] In the present study, most of the patients had a normal reproductive and menstrual history. The age at menarche of the patients varied from 12 to 16 years and age at menopause was between 43 and 55 years. Most of them consumed a vegetarian diet and none of them reported alcohol consumption. Thus, some genetic and environmental factors might be acting synergistically and are responsible for the high incidence of breast cancer in this area. Amritsar has many small-scale industries, such as textile processing, woolen, dyeing, electroplating, pharmaceutical, iron foundaries, pulp and paper mills, steel plants, dairy, and glass and plastic mills, and the area adjoining the city is mainly agricultural land, where the use of pesticides and agricultural chemicals is high. Heavy metal contamination has also been reported in agricultural products, soil, and water in and around Amritsar (www.punjabenvironment.com).

In the present study, the patients had much higher CIN than controls. Even patients with benign disease or at stage I had higher CIN than controls. But, the patients had variation in the level of CIN in PBLs with no apparent correlation with disease stage, as a stage I patient had up to 60% aberrant metaphases while a stage II patient had only 3.3% aberrant metaphases. The present study is in agreement with the previous reports on validity of cytogenetic assay for determination of frequency of CAs as a biomarker for cancer risk.[8–11] Such studies had been subject to criticism due to not accounting for the reverse causality bias, i.e. when the biomarker might be affected by the disease. But, the present study suggests an independence of this biomarker from disease stage. The inherent difference in the level of genomic instability might play a significant role in disease progression, patient tolerance for radiation and antineoplastic agents, and recurrence risk. Breast cancer (BRCA) proteins and their associated molecules (e.g., Fanconi anemia proteins, Ataxia telangiectasia mutated- Ras-associated diabetes (ATM- RAD complex) work in a network of connected biological complexes that encompass virtually all aspects of the cellular response to DNA damage during the S and G2 phases of the cell cycle.[30] Cells lacking these proteins fail to correct endogenous DNA damage during or after DNA synthesis. Individuals with mutation in BRCA or associated proteins show sensitivity to DNA cross-linking agents such as cisplatin and mitomycin C. Determination of the genomic instability level of individual patients before planning therapy may help avoid tissue and cellular damage by radiation and cancer chemotherapy drugs by permitting less-aggressive therapy of the sensitive patients.
